# *Lactobacillus plantarum* with Broad Antifungal Activity as a Protective Starter Culture for Bread Production

**DOI:** 10.3390/foods6120110

**Published:** 2017-12-11

**Authors:** Pasquale Russo, Clara Fares, Angela Longo, Giuseppe Spano, Vittorio Capozzi

**Affiliations:** 1 Department of Science of Agriculture, Food and Environment, University of Foggia, Via Napoli 25, 71122 Foggia, Italy; angela.longo@unifg.it (A.L.); giuseppe.spano@unifg.it (G.S.); vittorio.capozzi@unifg.it (V.C.); 2 Promis Biotech Via Napoli 25, 71122 Foggia, Italy; 3 Council for Agricultural Research and Economics—Research Centre for Cereal and Industrial Crops (CREA-CI), S.S.673 km 25.200, 71122 Foggia, Italy; clara.fares@crea.gov.it

**Keywords:** antifungal, bioprotection, bread, *Lactobacillus plantarum*, phenyllactic acid, *Aspergillus*, *Penicillium*, *Fusarium*

## Abstract

Bread is a staple food consumed worldwide on a daily basis. Fungal contamination of bread is a critical concern for producers since it is related to important economic losses and safety hazards due to the negative impact of sensorial quality and to the potential occurrence of mycotoxins. In this work, *Lactobacillus plantarum* UFG 121, a strain with characterized broad antifungal activity, was analyzed as a potential protective culture for bread production. Six different molds belonging to *Aspergillus* spp., *Penicillium* spp., and *Fusarium culmorum* were used to artificially contaminate bread produced with two experimental modes: (i) inoculation of the dough with a commercial *Saccharomyces cerevisiae* strain (control) and (ii) co-inoculation of the dough with the commercial *S. cerevisiae* strain and with *L. plantarum* UFG 121. *L. plantarum* strain completely inhibited the growth of *F. culmorum* after one week of storage. The lactic acid bacterium modulated the mold growth in samples contaminated with *Aspergillus flavus*, *Penicillium chrysogenum*, and *Penicillium expansum*, while no antagonistic effect was found against *Aspergillus niger* and *Penicillium roqueforti*. These results indicate the potential of *L. plantarum* UFG 121 as a biocontrol agent in bread production and suggest a species- or strain-depending sensitivity of the molds to the same microbial-based control strategy.

## 1. Introduction

Bread, obtained by baking a fermented dough of cereals flour, water, and other ingredients, is ancient and, due to its nutritional properties and low price, is a staple of many diets and an essential contributor of energy and nutritional intake in both developed and developing countries [1,2]. Microbial alteration of bread is a critical concern for bakeries, and it is mainly attributable to the development of spoilage molds. Apart from significant economic losses due to the negative impact on sensory properties, the occurrence of filamentous fungi poses a safety hazard for human health due to the potential ability of some fungal strains to produce mycotoxins [3,4]. Moreover, fungal spoilage control is critical for the extension of the shelf life of bakery goods, especially from an industrial perspective [5]. Traditionally, the shelf life of bread has been extended by the addition of chemical preservatives such as ethanol and weak organic acids, mainly propionic, sorbic, benzoic, and acetic acid and their salts [6]. As an alternative, physical methods such as microwave and infrared radiation and innovative packaging technologies have been exploited to reduce fungal developments in bakery products [7]. However, a strong societal demand, supported by public authorities, has urged more eco-friendly approaches mainly relying on the use of essential oils and antagonistic microorganisms as preservation tools [8]. In this context, lactic acid bacteria (LAB) have the greatest appeal as biocontrol agents due to their status of food-grade microorganisms [9,10]. The antifungal ability of some LAB strains is owed to the production of secondary metabolites, mainly including lactic acid and other organic acids, phenolic compounds, carbon dioxide, ethanol, hydrogen peroxide, fatty acids, acetoin, diacetyl, and cyclic dipeptides [11,12,13]. Moreover, synergistic interactions among different bioactive molecules could substantially increase the overall LAB antimicrobial activity [14]. Sourdough is a valuable and comprehensive source of antifungal and mycotoxin-controlling compounds synthesized by LAB during fermentation [15,16,17]. In the last few years, several LAB strains belonging to the species *Lactobacillus amylovorus*, *Lactobacillus reuteri*, *Lactobacillus brevis*, *Lactobacillus plantarum*, *Lactobacillus rossiae*, and *Lactobacillus paralimentarius* have been proposed as starter protective cultures to enhance the shelf life of bread [18,19,20,21,22]. Antifungal properties against bakery product spoilage molds have also been shown by *Propionibacterium* cultures [23]. Moreover, the ability of antagonistic yeasts to control fungal contamination in bread has been investigated in *Meyerozyma guilliermondii* and *Wickerhamomyces anomalus* [24,25]. In particular, *Penicillium roqueforti* was delayed until 14 days of storage in bread produced with a combination of these antifungal yeasts and of a specific *L. plantarum* strain [25]. Furthermore, proteinaceous compounds from different food matrices or legumes flour hydrolysates could be used as ingredients in the bakery industry to enhance the antifungal properties of sourdoughs [26,27].

In this work, a strain of *L. plantarum* previously characterized for its antifungal potential [28] was investigated for its ability to control the growth of six different species of filamentous fungi belonging to three different genera in artificially contaminated bread after one week of storage.

## 2. Materials and Methods 

### 2.1. Microbial Strains and Growth Conditions

*Lactobacillus plantarum* UFG 121 was available at the Laboratory of Industrial Microbiology of the University of Foggia (Foggia, Italy) and routinely grown in de Man Rogosa Sharpe (MRS) broth (Oxoid, Basingstoke, UK) at 30 °C for 24 h.

Six filamentous fungi, namely *Penicillium roqueforti* CECT 20508, *Penicillium expansum* CECT 2278, *Penicillium chrysogenum* CECT 2669, *Aspergillus niger* CECT 2805, *Aspergillus flavus* CECT 20802, and *Fusarium culmorum* CECT 2148 were provided by the Spanish Type Culture Collection (CECT, Paterna, Spain). Fungal cultures were plated on malt extract agar (Oxoid) and incubated at 24 °C for 5 days.

The commercial fresh yeast *Saccharomyces cerevisiae* “Lievital” (Lessafre, Marcq-en-Baroeul, France) was resuspended in sterile saline solution, streaked on plates of yeast extract, peptone, dextrose (YPD, Oxoid), and incubated at 30 °C for 48 h. Then, a single colony was resuspended in YPD broth and incubated at 30 °C.

### 2.2. Dough and Sourdough Preparation 

Cells at exponential phase of *S. cerevisiae* and *L. plantarum* UFG 121 were recovered by centrifugation (5000× *g*, 5 min), washed twice with sterile saline solution, and resuspended in sterile water. For sourdough preparation, microbial starters were inoculated at a concentration of about 10^8^ and 10^6^ cfu g^−1^ for *L. plantarum* and yeast respectively, in a mixture of wheat flour type “0” Manitoba (initial moisture of 11.9% *w*/*w*, supplied by Lo Conte, IPAFOOD, Ariano Irpino, Italy) and water (37.5% *w*/*w*) containing sucrose (6%), NaCl (3% *w*/*w*), and animal fats (3% *w*/*w*). A dough control sample was inoculated only with the commercial yeast at a final concentration of about 10^6^ cfu g^−1^. The fermentation was carried out at 30 °C for 18 h.

### 2.3. Bread Production

Bread was obtained in a pilot plant according to the methods ACC10-10B29 modified by Capozzi et al. [29]. Briefly, the sourdough was subject to kneading for 10 min, followed by fermentation for 90 min at room temperature. The sourdough was then aliquoted in samples of about 250 g each and fermented for another 90 min. Afterwards, samples were put into apposite shapes to attain specific dimensions (11 cm × 25 cm × 7 cm) and submitted to a final fermentation step of 90 min at room temperature. Bread was obtained after the dough was baked in an oven at 220 °C for 40 min. The bread was cut in half in order to repeat each experimental condition in duplicate.

### 2.4. Artificial Contamination of Bread

A preparation of fungal spores was obtained by brushing with a sterile swab the plate surface of each five-day-grown mold. Spores were resuspended in sterile distilled water and concentrated at 8 × 10^4^ spores mL^−1^. Artificially contaminated samples were obtained by spraying 15 mL of the spore solution on the surface of the bread. Samples were immediately packaged using polyethylene terephthalate bags and stored for 7 days at room temperature. At this time, the in vivo antagonistic activity against each tested mold was qualitatively determined by comparing the area contaminated by the spoilage fungi in bread fermented with the starter yeast or co-fermented with *L. plantarum* UFG 121. Results were expressed according to the following scale: no/low (−), moderate (+), high (+ +), and very high (+ + +) inhibition activity, if the area covered by each filamentous fungi in bread co-fermented with UFG 121 strain was reduced in the ranges 0–25%, 25–50%, 50–75%, and 75–100%, respectively.

### 2.5. Sensorial Quality

The sensorial analysis was carried out at our laboratory by a panel of six panelists before the artificial contamination of the samples with the spoilage molds. Panelists were previously trained in order to recognize and score the analyzed quality descriptors. Samples were coded with a random 3-digit number in order to minimize subjectivity. The sensorial attributes evaluated were overall appearance, aromatic profile, off odor, color, softness, and texture. Every attribute was scored on a 1–5 hedonic scale, where 1 = atypical, undesirable, and 5 = typical, desired. Sensorial trials were performed on six samples for each treatment.

### 2.6. Statistical Analysis

The quality descriptors analyzed in the sensorial trials were subjected to one-way analysis of variance (ANOVA). Pairwise comparison of treatment means was achieved by Tukey’s procedure, with a significance level of *p* ≤ 0.05, using the statistical software Past 3.0 (University of Oslo, Oslo, Norway).

## 3. Results

### 3.1. Production of Bread Co-Fermented with a Protective L. plantarum Strain

**Figure 1:**
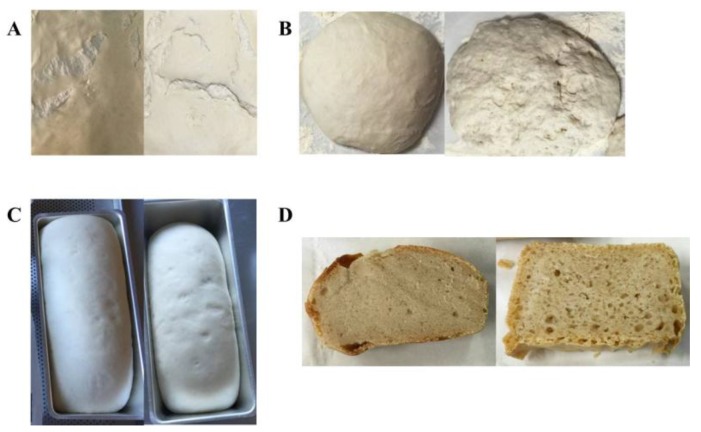
Dough fermented by *S. cerevisiae* “Lievital” (left pictures) or co-fermented with *L. plantarum* UFG 121 (right pictures) after (**A**) first fermentation step for 18 h at 30 °C; (**B**) kneading and shaping; (**C**) the final fermentation step for 90 min at room temperature; and (**D**) baking and cutting.

**Figure 2:**
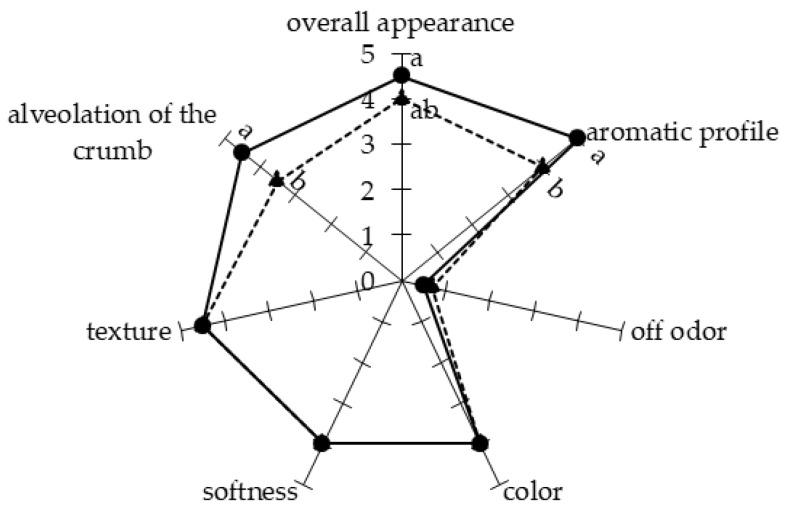
Sensory attributes of bread obtained by fermentation with *S. cerevisiae* “Lievital” (triangle, dashed line) or co-fermented with *L. plantarum* UFG 121 (circle, continuous line). Reported values are means of six independent replicates, and they are expressed by using a hedonic scale from 1 to 5 (1 = atypical, undesirable; 5 = typical, desired). Means with different letters are significantly different according to Tukey’s test (*p* ≤ 0.05).

Bread samples were produced by fermentation of the dough with a commercial yeast commonly used in breadmaking, or by its co-inoculation with *L. plantarum* UFG 121, a strain with a characterized antifungal activity [28]. A preliminary qualitative analysis was performed during the breadmaking process in order to detect if the employment of the protective strain *L. plantarum* UFG 121 as a starter culture could affect the bread production from a technological and/or sensorial point of view. As reported in Figure 1, some differences were observed among samples inoculated with the commercial yeast starter or co-inoculated with UFG 121 strain. In particular, after 18 h of the fermentation step, the dough was apparently softer when fermented with *S. cerevisiae* compared with the co-inoculation approach using the protective *L. plantarum* strain (Figure 1A). In contrast, the sourdough developed a more complex aromatic profile when co-fermented with UFG 121 strain. In agreement with this finding, after kneading the dough fermented using the yeast starter, the dough was more compact and elastic and therefore easier to break and to produce the desired shape. Co-fermentation with yeast and *L. plantarum* resulted in a sourdough with an inhomogeneous texture, which could be disadvantageous for subsequent processing (Figure 1B). However, no important differences were detected after the final fermentation, since both samples were well compacted and leavened with a smooth surface (Figure 1C). After all samples were baked, similar features in terms of color, softness, and texture were apparent. However, samples co-inoculated with *L. plantarum* UFG 121 presented more pronounced alveolation (Figure 1D and Figure 2). Moreover, sensorial analysis indicated the absence of off odors in both breads, while the aromatic profile of the bread inoculated with *L. plantarum* UFG 121 scored higher than the control bread. In general, the overall appearance of the bread obtained via UFG 121 co-fermentation was perceived as slightly better by the panelists.

### 3.2. Analysis of the Protective Potential of L. plantarum UFG 121 in Artificially Contaminated Bread

**Figure 3:**
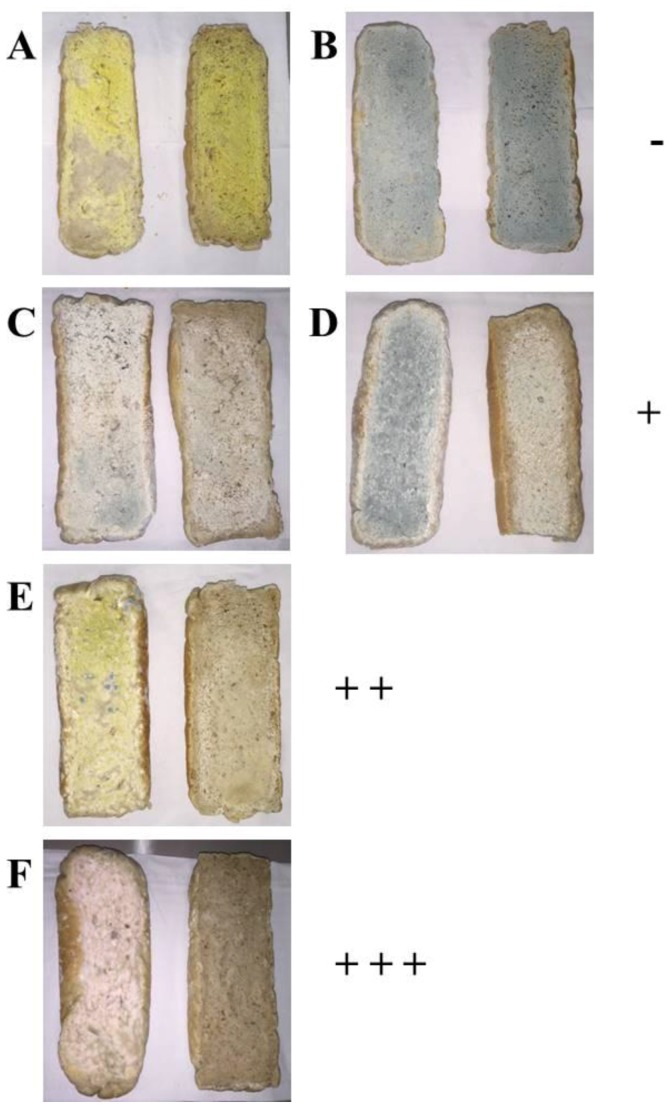
Bread obtained by fermentation with *S. cerevisiae* “Lievital” (left pictures) or co-fermented with *L. plantarum* UFG 121 (right pictures) after one week of storage at room temperature and artificially contaminated with *A. niger* CECT 2805 (**A**); *P. roqueforti* CECT 20508 (**B**); *P. chrysogenum* CECT 2669 (**C**); *P. expansum* CECT 2278 (**D**); *A. flavus* CECT 20802 (**E**); *F. culmorum* CECT 2148 (**F**). Antifungal activity was expressed as no/low (−), moderate (+), high (+ +), and very high (+ + +), when the contaminated area was reduced in the ranges 0–25%, 25–50%, 50–75%, and 75–100%, respectively.

With the aim to evaluate the potential of *L. plantarum* UFG 121 as a culture protective against typical molds on bakery products, bread samples were artificially contaminated by spraying a concentrated spore suspension onto the bread slices. After one week of storage, the in vivo antagonistic activity against each tested mold was qualitatively determined by comparing the area covered by the spoilage fungi in the two tested experimental modes: (1) bread fermented with the commercial *S. cerevisiae* and (2) co-fermented with *L. plantarum* UFG 121 (Figure 3). As shown in Figure 3, after one week of storage the surface of the control bread artificially contaminated were always wholly covered by the molds. In contrast, different scenarios were observed when bread samples were co-fermented with *L. plantarum* UFG 121. In particular, no inhibition was found in bread samples artificially inoculated with *A. niger* and *P. roqueforti* that appeared completely contaminated by both the molds (Figure 3A,B). A moderate in vivo antagonistic activity was detected against *P. chrysogenum* and *P. expansum* whose growth was limited in samples obtained with UFG 121 strain (Figure 3C,D). In contrast, a higher protective effect was observed in samples contaminated by *A. flavus* in which only approximately 20% of the bread surface was covered by the spoilage (Figure 3E). Interestingly, no development of *F. culmorum* was observed, suggesting that the employment of *L. plantarum* UFG 121 during bread fermentation was a successful strategy to thoroughly inhibit *F. culmorum* growth (Figure 3F).

## 4. Discussion

In the present study, six different molds belonging to *Aspergillus* spp., *Penicillium* spp., and *Fusarium culmorum* were used to artificially contaminate bread produced by fermentation of the dough with a commercial *S. cerevisiae*, or by its co-inoculation with *L. plantarum* UFG 121. The fungal strains were selected because they are representative molds of bread spoilage [30] and they have an ability to produce mycotoxins. In particular, *A. flavus* CECT 20802 produced aflatoxins B1, B2, M1, M2; *F. culmorum* CECT 2148 fusarine C; *P. roqueforti* CECT 20508 was able to synthesize PR toxin; while *P. expansum* CECT 2278 was responsible for the production of patulin and citrinin. In the present study, *L. plantarum* UFG 121, previously characterized for its broad antifungal activity against these mold strains [28], was investigated as a protective culture for bread production.

The employment of LAB strains with antagonistic activity has been widely proposed as an innovative green strategy to fight spoilage filamentous fungi in order to enhance the shelf life of bakery products [24,31,32,33]. In general, the antifungal effect has been attributed to the production of some secondary metabolites during the fermentation of sourdough [11]. Therefore, according to previous studies [24,34], in this work, a fermentation step of 18 h was performed in order to allow the UFG 121 strain to enrich the sourdoughs with bioactive antifungal compounds. However, differences between the control samples and the bread obtained by co-fermentation with *L. plantarum* UFG 121 were qualitatively detected as the bread was made that might have affected the production process. Nonetheless, as previous reported by Coda et al. [25], co-fermentation of the dough with yeast and selected LAB resulted in bread showing good chemical and textural features, including elasticity, color, and alveolation. Moreover, an improvement in terms of complexity of the aromatic profile was noted in the bread co-fermented with UFG 121. In agreement with this finding, Makhoul et al. [35], analyzing pro-technological microbe/matrix interactions during food fermentation, reported a greater impact of the microbial fraction on the volatile organic compounds of bread. However, the present work is only a preliminary study that should be further complemented by the analytical determination of the main physico-chemical parameters as well as the impact of a protective LAB culture on the organoleptic profile of the bread [36,37].

In our previous study, we found that the preservative potential of *L. plantarum* UFG 121 was mainly due to the production of lactic acid and phenyllactic acid [28]. Phenyllactic acid production by *L. plantarum* has been linked to the antifungal activity against fungal strains isolated from bakery products belonging to species of *Aspergillus*, *Penicillium*, and *Fusarium* [38]. Similarly, an increase in the shelf life of bread obtained by fermentation with *L. plantarum* CRL 778 and artificially contaminated with *Penicillium* spp. has been found to be related to the synthesis of acetic and phenyllactic acid as well as lactic acid [20]. Moreover, organic acids including phenyllactic acid from a strain of *L. amylovorus* have been found to be responsible for a higher shelf life in gluten-free breads [21]. Lactic acid, phenyllactic acid, and two cyclic dipeptides found in sourdoughs fermented by *L. plantarum* FST 1.7 have been identified as the main antifungal compounds able to retard the growth of *F. culmorum* and *Fusarium graminearum* on bread [18].

In a similar way, in the present study, *F. culmorum* CECT 2148 was completely inhibited in bread fermented by *L. plantarum* UFG 121. The in vitro assays performed suggest that CECT 2148 was the most sensitive mold when exposed to UFG 121 cell-free supernatant [28]. Moreover, this result has been confirmed in situ, since fermentation by UFG 121 and artificial contamination with CECT 2148 (after thermal stabilization) increased the shelf life of an oat-based formulation from less than one week to the second week of cold storage, indicating that a strong bioprotection could be provide by antifungal compounds produced in a 16 h fermentation step [28]. In contrast, fermentation with *L. plantarum* UFG 121 had no effect in countering the growth of *P. roqueforti* CECT 20508 and *A. niger* CECT 2805, but reduced bread contamination by the remaining tested molds to different extents. Interestingly, these results were only partially predicted by the in vitro assays [28], suggesting that interactions with the commercial yeast, process parameters, and/or the food matrix might modulate the antagonistic activity of selected LAB against fungal strains. Therefore, further investigations are required to establish the effectiveness of the antifungal compounds, their synergistic interactions, and the complex microbial and physico-chemical relationships occurring in the food environment.

## 5. Conclusions

In recent years, several studies have aimed to enhance the shelf life and safety of bakery products by analyzing the antifungal potential of protective microbial starter cultures and the corresponding sourdough. However, most of these works have been performed using only one or a few mold strains as fungal indicators. In this study, although with a preliminary experimental plan, we analyzed the in situ bioprotection effectiveness of a *L. plantarum* strain against a representative panel of six different mold species belonging to three different genera generally recognized as being mainly responsible for the natural contamination of bakery foods. Our results indicated in these molds a species- or strain-dependent sensitivity to the same microbial-based control strategy, suggesting that a new generation of mixed starter protective cultures, active against different fungal species, might be better able to globally extend the shelf life of baked goods.
